# Monocyte and macrophage differentiation: circulation inflammatory monocyte as biomarker for inflammatory diseases

**DOI:** 10.1186/2050-7771-2-1

**Published:** 2014-01-07

**Authors:** Jiyeon Yang, Lixiao Zhang, Caijia Yu, Xiao-Feng Yang, Hong Wang

**Affiliations:** 1 Department of Pharmacology, Centers for Metabolic Disease Research, Cardiovascular Research, and Thrombosis Research, Temple University School of Medicine, Philadelphia, PA 19140, USA

**Keywords:** Monocytes, Inflammatory diseases

## Abstract

Monocytes express various receptors, which monitor and sense environmental changes. Monocytes are highly plastic and heterogeneous, and change their functional phenotype in response to environmental stimulation. Evidence from murine and human studies has suggested that monocytosis can be an indicator of various inflammatory diseases. Monocytes can differentiate into inflammatory or anti-inflammatory subsets. Upon tissue damage or infection, monocytes are rapidly recruited to the tissue, where they can differentiate into tissue macrophages or dendritic cells. Given the rapid progress in monocyte research from broad spectrum of inflammatory diseases, there is a need to summarize our knowledge in monocyte heterogeneity and its impact in human disease. In this review, we describe the current understanding of heterogeneity of human and murine monocytes, the function of distinct subsets of monocytes, and a potential mechanism for monocyte differentiation. We emphasize that inflammatory monocyte subsets are valuable biomarkers for inflammatory diseases, including cardiovascular diseases.

## Introduction

The primary role of monocytes was considered to sense the environment and replenish the pool of tissue macrophages and dendritic cells. Recent advances in immunology research have discovered that monocytes are heterogenic and can be divided into three subsets based on specific surface markers and that each subset displays specific functions. During steady state, circulating monocytes have a half-life of about one to three days, and maintain a steady composition of monocyte subsets.

Identified monocyte subsets exhibit distinct pathophysiological roles. Classical inflammatory monocytes are equipped with a set of Toll-like receptors (TLRs) and scavenger receptors, recognizing pathogen-associated molecular patterns (PAMPs) and removing microorganisms, lipids, and dying cells via phagocytosis. They produce effector molecules such as cytokines, myeloperoxidase and superoxide, and initiate inflammation [1].

Inflammatory monocytes selectively traffic to the sites of inflammation, produce inflammatory cytokines and contribute to local and systemic inflammation [2]. They are highly infiltrative and can be differentiated into inflammatory macrophages, which remove PAMPs and cell debris. In steady state, the patrolling anti-inflammatory monocytes patrol the vasculature to monitor PAMPs and become tissue resident macrophages. During inflammation, they differentiate into anti-inflammatory macrophages, which repair damaged tissues [3].

Murine monocyte subset classification and their functional determinations have been consistent and well accepted [4]. However, classification of human monocyte subsets in relevance to their inflammatory or anti-inflammatory functional phenotypes remains partially undefined. Here, we intend to review the current understanding regarding monocyte heterogeneity, and to integrate the knowledge of murine and human monocyte classification.

### Monocytosis and heterogeneous monocytes

It was first reported in the 1970s that monocytes increase proliferative activity in bone marrow (BM) in response to inflammatory stimuli, leading to monocytosis, [5] a clinical condition reflecting an increased number of circulating monocytes.

Emerging clinical analysis revealed a higher prevalence of monocytosis in cardiovascular diseases (CVD) (Table 1). Monocyte count is increased in acute myocardial infarction (AMI) patients compared to patients with stable coronary arterial disease (CAD) [6]. Peripheral monocytosis is associated with left ventricular (LV) dysfunction and LV aneurysm, suggesting a possible role of monocytes in the development of LV remodeling after reperfused AMI [7]. Monocytosis is also associated with reduced high-density lipoprotein (HDL) levels and impaired renal function in CAD patients [8]. It has been demonstrated that monocyte count is a better independent risk factor of CVD than several conventional risk factors such as C-reactive protein (CRP), inflammatory cytokine interleukin-6 (IL-6), fibrinogen, hypertension, and cigarette smoking [9]. The treatment of coronary arterial disease patients with pravastatin, a cholesterol lowering medication, for 6 months reduces plaque volume and monocyte count, implying that monocytosis is a potential target for coronary atherosclerotic regression [10].

**Table 1:** Monocytosis in human disease

**Disease**	**Group comparison**	**Monocyte counts comparison**	**PMID #**
CVD	HDL, 2 nmol/L vs. <1 nmol/L	3.65 × 10^8^ vs. 4.5× 10^8^ cells/dL	18629357
AMI	CT vs. AMI	4.97 × 10^8^ vs. 7.93 × 10^8^ cells/dL	23455782
AMI	5 h AMI vs. 1–2 day AMI	4.56 × 10^8^ vs. 7.11 × 10^8^ cells/dL	11788214
	None vs. Pump failure	6.05 × 10^8^ vs. 9.41 ×10^8^ cells/dL	
	None vs. LV aneurysm	6.82 × 10^8^ vs. 8.61 × 10^8^ cells/dL	
CKD	CKD without CVD vs. CKD with CVD	5.71 × 10^8^ vs. 6.97 × 10^8^ cells/dL	18160960
AMI	LVF recovery vs. LVF no recovery	6.42 × 10^8^ vs. 10.13 × 10^8^ cells/dL	17652884
CAD	Healthy vs. CAD vs. AMI	5.17 × 10^8^ vs. 5.42 × 10^8^ vs. 6.72 × 10^8^ cells/dL	16612453

Circulating total MC counts were examined in human disease as indicated. Comparisons were made between described groups in *CVD*, and MC counts. We used PMID # to cite individual manuscripts reporting these studies. *AMI*, acute myocardial infarction; *CAD*, coronary arterial disease; *CKD*, chronic kidney disease; *CK*, creatine kinase; CRP, C-reactive protein; CVD, cardiovascular disease; *LVF*, left ventricular failure. *PMID*, PubMed Identification.

Following the defining of monocytosis, reduced phagocytic capacity of monocytes was found in patients with rheumatoid arthritis and cutaneous vasculitis [11]. Patients with lymphopenia have suppressor monocytes, which are unable to activate T-cells [12]. These findings suggested the existence of heterogeneous monocyte populations. Further studies for different functional properties of such populations identified that CD16 (Leu-11), a Fc receptor (FcR) as it binds to the Fc region (constant region) of antibody, is expressed on the surface of monocytes and correlated with atherosclerosis and CVD in patients and an inflammatory phenotype in cultured monocytes and circulating monocytes [13]. The CD16^+^ monocytes has been considered an inflammatory monocyte subset in humans [14].

### Mouse monocyte subsets

Monocyte subsets in mice were first identified by differential expression of chemokine receptors CCR2. CCR2^+^ subset shows higher migratory and infiltration capacity than CCR2^-^ subset and was initially considered as murine inflammatory monocyte [15]. Later on, mouse monocyte subsets are characterized by differential expression of an inflammatory monocyte marker Ly6C (Gr1). It is now accepted that mouse monocyte subsets are grouped as Ly6C^+^ (further divided as Ly6C^high^ + Ly6C^middle^) and Ly6C^-^ (also called Ly6C^low^) monocyte subsets based on expression levels of Ly6C on cell surface (Table 2). The surface markers and chemokine receptors for Ly6C^+^ subsets are CD11b^+^CD115^+^ and CCR2^high^CX3CR1^low^. Whereas, the surface markers and chemokine receptors for Ly6C^-^ monocytes are CD11b^+^CD115^+^ and CCR2^low^CX3CR1^high^[16].

**Table 2:** Markers and functions of MC subsets in human and mouse

**Species**	**Subsets**	**Surface markers**	**% in MNC**	**Chemokine receptors**	**Functions**
Human	Classical	CD14^++^CD16^-^	80-95	CCR2^high^CX3CR1^low^	Phagocytosis
	Intermediate	CD14^++^CD16^+^	2-11	CCR2^mid^CX3CR1^high^CCR5^+^	Pro-inflammatory
	Non-classical	CD14^+^CD16^++^	2-8	CCR2^low^CX3CR1^high^	Patrolling
Mouse	Ly6C^high^ (Ly6C^+^)	CD11b^+^CD115^+^Ly6C^high^	40-45	CCR2^high^CX3CR1^low^	Phagocytosis & Pro-inflammatory
	Ly6C^middle^ (Ly6C^+^)	CD11b^+^CD115^+^Ly6C^middle^	5-32	CCR2^high^CX3CR1^low^	Pro-inflammatory
	Ly6C^low^ (Ly6C^-^)	CD11b^+^CD115 ^+^Ly6C^low^	26-50	CCR2^low^CX3CR1^high^	Patrolling; tissue repair

Human MCs are divided into three subsets based on the cell surface expression of CD14 and CD16. CD14^++^CD16^-^ MCs, also called the classical MC, are the most prevalent MC subset in human blood and express high level of CCR2. The CD14^++^CD16^+^ MCs are intermediate MC which contribute significantly to atherosclerosis. The CD14^+^ CD16^++^ MCs are referred to as non-classical monocytes which perform a *in vivo* patrolling function. Mouse MCs are divided into two subsets based on their cell surface expression of Ly6C. The Ly6C^high^ and Ly6C^middle^ subsets perform pro-inflammatory functions and express high level of CCR2, which is considered the counterpart of human classical MCs. The Ly6C^low^ subsets express low level of *CCR*2, majorly patrol along the vascular endothelium and are involved in tissue repair, functionally similar to human non-classical MCs. *CD*, cluster of differentiation; *CCR*2, chemokine (C-C motif) receptor 2; *CX3CR1*, CX3C chemokine receptor 1; *Ly6C*, lymphocyte antigen 6 complex.

### Functional properties of mouse monocyte subsets

**Figure 1:**
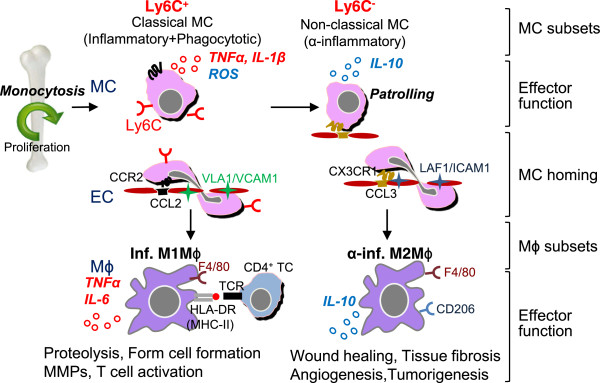
**Human MC and M**ϕ **differentiation**, **and distinct subset functions.** Human CD14^++^CD16^-^ classical MCs leave the bone marrow in a CC-chemokine receptor 2 (CCR2)-dependent manner. In the steady state, classical MCs can differentiate into intermediate MCs, then differentiate into patrolling non-classical MCs in circulation. Classical MCs have a high antimicrobial capability due to their potent capacity of phagocytosis, and secrete ROS and IL-10 upon LPS stimulus, whereas intermediate and non-classical MCs secrete inflammatory cytokines, TNFα and IL-1β upon inflammatory stimulation. During inflammation, classical and intermediate MCs are tethered and invade tissue by interaction of complementary pair CCR2/CCL2(MCP1) or/and CCR5/CCL5(RANTES) in a VLA1/VCAM1 dependent manner. MCs then mature to M1Mϕ in tissue and present self-antigen via MHC-I/II to TCR leading to TC activation. Non-classical MCs patrol the vessel wall and invade by interaction of complementary pair of CX3CR1/CCL3 via LAF/ICAM1-dependent manner. TC, T cell; MC, monocyte; Mϕ, macrophage; EC, endothelial cells; inf., inflammatory; α-inf. Anti-inflammatory; TCR, T cell receptor; HLA-DR, human leukocyte antigen DR (a major histocompatibility complex class II (MHC-II)).

As shown in Figure 1, mouse Ly6C^+^ monocytes have a high antimicrobial capability due to their potent capacity for phagocytosis, secrete ROS, TNFα, nitric oxide, IL-1β, little IL-10 upon bacterial infection [17] and large amount of type 1 interferon (IFN) in response to viral ligands [18]. CCR2-CCL2 signaling in Ly6C^+^ monocytes alters the conformational change of VLA-4 (α4β1 integrin), the ligand for VCAM-1, leading to high affinity interaction and monocyte transmigration (Figure 1). In vascular inflammation, Ly6C^+^ monocytes are preferentially recruited into inflamed tissue via interaction of chemokine receptor CCR2 [19] and more likely to mature to inflammatory M1 macrophages, which are distinguished by secretion of pro-inflammatory cytokine, TNFα, and IL-6 and contribute to tissue degradation and T cell activation.

In steady state, Ly6C^+^ monocytes differentiate into Ly6C^-^ monocytes in the circulation. This subset patrols the luminal side of endothelium of small blood vessels and bind to endothelium by chemokine receptor CX3CR1 via LAF-1/ICAM1-dependent manner. The patrolling behavior of monocytes may be due to low levels expression of adhesion molecules. Ly6C^-^ monocytes secrete anti-inflammatory cytokine, IL-10 upon *in vivo* bacterial infection. In vascular inflammation, Ly6C^-^ monocytes are recruited to tissue and more likely to differentiate into M2 macrophages, which secrete anti-inflammatory cytokine and contribute to tissue repair (Figure 1) [20].

Recruited monocytes/macrophages may emigrate from vessels and enter lymph nodes, which are associated with regression of atherosclerotic lesions [21]. Notably, CD62L (L-selectin) expressed by leukocytes, including Ly6C^+^ monocytes, is important for circulation to lymph nodes through high endothelial venules (HEV) [15]. Chemokine receptor CCR7 and CCR8, responsible for lymph node traffic, were selectively expressed by Ly6C^middle^ monocytes [22].

### Human monocyte subsets

Because CD14 is abundantly expressed on the surface of human monocytes and macrophages, it is used to mark human monocytes. Compared to CD14^+^CD16^-^ (also described as CD14^bright^CD16^-^) monocytes, the human CD14^+^CD16^+^ (also described as CD14^dim^CD16^+^) monocyte subset has reduced phagocytic capacity, produces less reactive oxygen species (ROS) and expresses lower levels of CCR2, a chemokine receptor mediating monocyte chemotaxis during inflammation and higher levels of CX3CR1, a chemokine receptor mediating resident monocyte accumulation [23]. Because the chemokine expression pattern implies CD16^+^ monocyte has an anti-inflammatory function, there was confusion on the characterization of human monocyte subsets [23]. However, CD14^+^CD16^+^ monocytes also express CCR2 and are associated with Crohn’s disease [24] and CVD [25]. Several earlier clinical studies used CD14^+^CD16^+^ as the inflammatory monocyte criteria and established the association of increased levels of CD14^+^CD16^+^ monocyte in human inflammatory diseases, including rheumatoid arthritis, coronary arterial disease, atherosclerosis, hemophagocytic syndrome, and Crohn’s disease (Table 3). Moreover, circulating CD16^+^ monocyte levels are positively correlated with levels of atherogenic lipids [26] and plaque vulnerability [27], whereas it is negatively correlated with cardiac function such as left ventricular (LV) ejection fraction after AMI [28]. Significant increases in CD16^+^ monocyte levels have been described in human chronic pathologies in obesity as well [29]. In the same study, several groups reported differential expression of CD14^dim^ and CD14^high^ within CD16^+^ monocytes [26,30], which was related to distinct functional properties of the chemokine receptor expression pattern [31]. A panel of leading experts in monocyte biology proposed consensus nomenclature for human monocyte subsets in 2010, and classified human monocytes subsets as classical monocytes (CD14^++^CD16^-^), intermediate monocytes (CD14^++^CD16^+^), and non-classical monocytes (CD14^+^CD16^++^) [32].

**Table 3:** Frequency of two MC subsets in human diseases

**Disease**	**CD14**^**++**^**CD16**^**- **^**(classical, phagocytic)**	**CD14**^**+**^**CD16**^**+ **^**(Non-classical, inflammatory)**	**Functional changes associated with CD14**^**++**^**CD16**^**+ **^**MC expansion**	**PMID** #
Rheumatoid Arthritis	No change	2.2% ↑	HLA-DR and CCR5↑ Counts of tender/swollen joints↑ Rheumatoid factors ↑	12384915
CAD		2.2% ↑	Serum TNFα ↑	15269840
CAD		8% ↑	Plaque vulnerability↑	20684824
Atherosclerosis	8% ↓	8% ↑		19461894
Hemophagocytic syndrome		31% ↑	Serum TNFα & IL-6↑	17619880
Crohn’s disease		5.7% ↑		17260384
Tumor/haematological malignancy		13.3% ↑		10209505

Circulating classical (CD14^++^CD16^-^, also described as CD14^bright^CD16^-,^ phagocytic) and non-classical (CD14^+^ CD16^+^, also described as CD14^bright^CD16^+^, inflammatory) MC counts were examined in human disease as indicated. The percentage change of MC subsets and some functional measurements are recorded. We used PMID # to cite individual manuscripts reporting these studies. *MC*, monocyte; *AMI*, acute myocardial infarction; *CAD*, coronary arterial disease; *CKD*, chronic kidney disease; *HLA*-*DR*, human leukocyte antigen DR (*MHC*-II, major histocompatibility complex class II); *TNFα*, tumor necrosis factor α; *IL*-6, interleukin 6; ↑, increase.

As indicated in Table 2, CD14^++^CD16^+^ monocytes express CCR2 and selectively CCR5, which react with macrophage inflammatory protein-1α (MIP-1α), a chemotactic chemokine for macrophages and CCL5 (termed regulated on activation, normal T cell expressed and secreted, RANTES). CCR5, known as a co-receptor for human immunodeficiency virus entry into macrophages, is also associated with CVD [31,33]. CD14^++^CD16^-^ monocytes express highest levels of CCR2 and CD14^+^CD16^++^ monocytes express highest levels of CX3CR1 [31].

Although much more evidence supports that Ly6C^+^ and CD14^+^CD16^-^ classical monocytes are pro-inflammatory monocytes, their high expressions of CD62L imply a possible role of lymph node migration and differentiate into a variety of macrophages and dendritic cell subtypes that could inhibit immune response [34]. Understanding the functions of subsets provides an insight in extrapolating results from clinical studies of inflammatory monocytosis found in patients’ blood with various inflammatory diseases.

### Functional properties of human monocyte subsets

**Figure 2:**
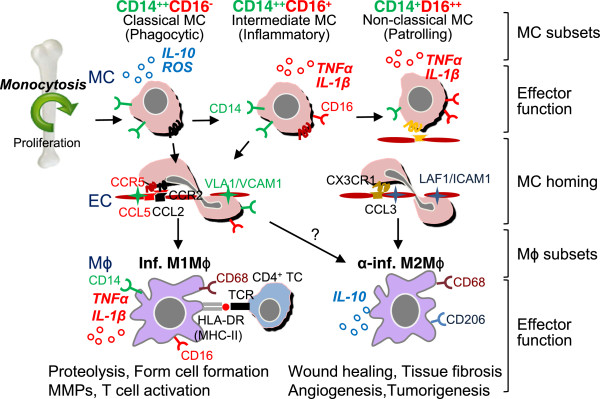
**Murine MC and M**ϕ **differentiation**, **and distinct subset functions.** Mouse Ly6C + MCs leave the bone marrow in a CC-chemokine receptor 2 (CCR2)-dependent manner. In the steady state, Ly6C + MCs differentiate into Ly6C- MCs in the circulation. Ly6C- MCs are recruited into normal tissue by interaction of complementary pair CX3CR1/CCL3 via a LAF/ICAM1-dependent manner and become tissue resident Mϕ/DCs. Ly6C + MCs have a high antimicrobial capability due to their potent capacity for phagocytosis, and secrete ROS, TNFα, and IL-1β, whereas Ly6C- MCs secrete anti-inflammatory cytokine IL-10 upon *in vivo* bacteria infection. In vascular inflammation, Ly6C + MCs are tethered and invade tissue by interaction complimentary pair of CCR2/CCL2(MPC-1) via a VLA-1/VCAM1-dependent manner, then mature to inflammatory M1Mϕ. M1Mϕ are distinguished by secretion of pro-inflammatory cytokines, TNFα and IL-6 and contribute to tissue degradation and T cell activation. Ly6C- MCs are recruited to tissue and differentiate into M2Mϕ, which secrete anti-inflammatory cytokine and contribute to tissue repair. *TC*, T cell; *MC*, monocyte; *M*ϕ, macrophage; *EC*, endothelial cells; *DC*, dendritic cell; inf., inflammatory; α-inf. Anti-inflammatory; *TCR*, T cell receptor; HLA-DR, human leukocyte antigen DR (a major histocompatibility complex class II (MHC-II)).

As shown in Figure 2, human CD14^++^CD16^-^ classical monocytes express high levels CCR2 and CD62L (L-selectin), and low levels of CX3CR1. Their major function is phagocytosis. They are phagocytic, exhibit high peroxidase activity, and produce high levels of IL-10 and low levels of TNF-α in response to LPS [23,35]. Gene expression profiling analysis indicates that human classical monocytes preferentially express genes involved in angiogenesis, wound healing, and coagulation [36]. Human CD14^++^CD16^+^ intermediate monocytes display inflammatory function. This subset has low peroxidase activity but higher capacity to produce and release IL-1β, and TNFα in response to LPS [35]. Gene signature links CD14^++^CD16^+^ monocytes to antigen presentation and T cell activation (Figure 2) [36]. During inflammation, classical and intermediate monocytes are tethered and invade tissue by interaction of complementary pair of CCR2/CCL2 (termed monocyte chemoattractant protein, MCP) or/and CCR5/CCL5 in a Very Late Activation Antigen-1 (VLA1)/VCAM1 dependent manner.

Human CD14^+^CD16^++^ non-classical monocytes, patrol the vessel wall and invade by interaction of complementary pair of CX3CR1/CCL3 via the Leu-CAM family integrin lymphocyte functional antigen-1 (LFA-1)/ICAM1-dependent manner (Figure 2). This subset releases IL-1β, and TNFα in response to DNA, RNA particles, implicating the pathological role in autoimmune disease such as rheumatoid arthritis [35].

In human CVD and inflammatory conditions, inflammatory intermediate CD14^++^CD16^+^ monocyate is increased (Tables 3 & 4). However, the change of CD14^+^CD16^++^ non-classical monocyte count is inconsistent; CD14^+^CD16^++^ monocyte count is increased in chronic kidney disease (CKD), abdominal aortic aneurysms (AAA), sepsis, hepatitis B, human immunodeficiency virus (HIV) infection and tuberculosis, but decreased in congestive heart failure, stroke and sepsis. It was suggested that CD14^++^CD16^-^ and CD14^++^CD16^+^ monocytes resemble mouse Ly6C^+^ inflammatory monocyte subset, whereas CD14^+^CD16^++^ monocytes may resemble Ly6C^-^ anti-inflammatory monocytes and have potential role of patrolling vascular endothelium [23]. However, some studies emphasize the inflammatory role of CD14^+^CD16^++^ cells because of the production of inflammatory cytokines. Nevertheless, much attention has been focused on the changes of CD14^++^CD16^+^ intermediate monocyte count in patients with inflammatory diseases. Since CD14^++^CD16^+^ monocyte count increases are consistently associated with human inflammatory disease (Tables 3 & 4), it is a sufficient biomarker of chronic and acute inflammatory diseases.

**Table 4:** Frequency of three monocyte subsets in different diseases

**Disease**	**CD14**^**++**^**CD16**^**- **^**(Classical, phagocytic)**	**CD14**^**++**^**CD16**^**+ **^**(Intermediate, inflammatory)**	**CD14**^**+ **^**CD16**^**++ **^**(Non**-**classical, patrolling)**	**Functional change associated with CD14**^**++**^**CD16**^**- **^**MC expansion**	**PMID #**
Congestive HF		6.4%↑		CD143 (ACE) ↑, Creatine↑, GFR↓, albumin↓	20364047
CKD		42 → 70 cells/μl	55 → 130 cells/μl		20943670
RA		5%↑		Th17 cells expansion	22006178
AAA		2.24%↑	1.9%↑		23348634
Stroke		3%↑	3%↓		19293821
HIV-2		7%↑		Myeloid dendritic cell depletion	23460749
Sepsis	No change	11.5%↑	6%↑	Phagocytosis↓, CD86↑, HLA-DR↓, IL-1β↓, IL-10↑	12028567
Sepsis	9.5%↓	12%↑	3.4%↓	HLA-DR↓, TNFα & IL-1β ↓,IL-10↑	19604380
Hepatitis B	6.2%↓	3.3%↑	2.5%↑	HLA-DR↑,TNF α ↑, IL-6↑, IL1β ↑, Th17 cells expansion	21390263
HIV	2.5%↓	3%↑	3%↑	CD163(scavenger receptor)↑	21625498
Denque fever	12 ~ 18%↓	3 ~ 7%↑		HLA-DR ↓, ICAM ↑, serum TNFα↑, IL-18 ↑, IFNγ ↑ ,	20113369
Tuberculosis	10%↓	9%↑	13%↑	TNFα ↑, apoptosis↑, Il-10↓	21621464

Circulating classical (CD14^++^CD16^-^, also described as CD14^+^ CD16^-^, phagocytic), intermediate (CD14^++^CD16^+^, also described as CD14^+^ CD16^+^, inflammatory) and non-classical (CD14^+^ CD16^++^, also described as CD14^dim^CD16^+^, patrolling) MC counts were examined in human disease as indicated. The percentage change of monocyte subsets and some functional measurements are recorded. We used PMID # to cite individual manuscripts reporting these studies. *ACE*, angiotensin converting factor; *GFR,* glomerular filtration rate; CD86, co-stimulatory molecule, *HLA-DR,* human leukocyte antigen DR (MHC-II, major histocompatibility complex class II); *RA,* rheumatoid arthritis; *AAA,* abdominal aortic aneurysms; *HF,* heart failure; *CKD,* chronic kidney disease; *GFR,* glomerular filtration rate; *HIV,* human immunodeficiency virus; ↑, increase; ↓, decrease; →, change to.

### Monocyte differentiation

Monocytes are differentiated from the committed precursor termed macrophage-DC precursor (MDP) mainly resident in bone marrow and differentiate into either dendritic cells or macrophages. They consist of two main subpopulations: CX3CR1^high^CCR2^low^Ly6C^-^ and CX3CR1^low^CCR2^high^Ly6C^+^. However, it is unclear whether Ly6C^-^ monocyte is differentiated from CX3CR1^low^CCR2^high^Ly6C^+^ or directly from bone marrow MDP. After maturation, Ly6C^+^ monocytes leave bone marrow and enter into the blood stream via CCR2 mediated migration [37]. After leaving the bone marrow, mouse Ly6C^+^ monocytes differentiate into Ly6C^-^ monocytes in circulation [38]. A recent monocyte fate mapping study strongly supported that in the steady state, Ly6C^+^ monocyte is the obligatory precursor for generation and lifespan control of Ly6C^-^ monocyte in the bone marrow, peripheral blood and spleen. In a competitive setting of mixed CCR2-proficient (CD45.1) and CCR2-deficient (CD45.2) (Ly6C^+^ monocytes are reported to be selectively reduced) BM chimeras, CD45.1^+^ WT Ly6C^-^ monocytes outcompeted their CD45.2 mutant Ly6C^-^ counterparts [39]. In the same study, Ly6C^+^ monocytes restored regained Ly6C^-^ half-life and the population.

Similarly, in human monocyte differentiation, it is accepted that CD14^++^ classical monocytes leave bone marrow and differentiate into CD14^++^CD16^+^ intermediate monocytes and sequentially to CD14^+^CD16^++^ non-classical monocytes in peripheral blood circulation [40].

### Monocyte to macrophage differentiation

CCR2^hi^Ly6C^+^ inflammatory and CCR^low^Ly6C^-^ resident monocytes are generally thought to preferentially differentiate into M1 inflammatory and M2 anti-inflammatory macrophages, respectively, during early inflammation [20]. Ly6C^+^ monocytes dominate the early phase of myocardial infarction and exhibit phagocytic, proteolytic, inflammatory function and digest damaged tissue. On the other hands, Ly6C^-^ monocytes, recruited at later phase of inflammation, attenuate inflammatory properties and differentiate toward M2 macrophages and contribute to angiogenesis, genesis of my fibroblasts, and collagen deposition (Figure 1). It is possible that monocytes and macropahge are highly plastic and can be crossly differentiated into different subsets in response to environment changes. Several studies revealed “unusual” cascades of monocytes to macrophage transition: *1*) Infiltrated Ly6C^+^ monocytes in inflamed skeletal muscle or brain tissues acquire phenotypic features of anti-inflammatory monocytes by down-regulating Ly6C expression, thereby displaying anti-inflammatory M2 macrophages function; [41,42]*2*) Ly6C^middle^ monocytes emigrate to lymph nodes via CCR7 and CCR8 and differentiate into dendritic cells; [22,43]*3*) During steady state, Ly6C^+^ monocytes are recruited to healthy lamina propria and differentiate into tissue resident CX3CR1^high^ macrophages; [44]*4*) M2 macrophages are generated by alternative activation of tissue-resident macrophages rather than recruited monocytes during infection with *Litomosoides sigmodontis*; [45] and *5*) Inflammatory monocyte recruitment to allergic skin is essential to alleviate allergic inflammation in order to acquire an anti-inflammatory M2 phenotype via basophil-derived IL-4 [46]. These findings demonstrated the multiple capacities of monocytes to differentiate into either regulatory or inflammatory mature macrophages/dendritic cells.

### Inflammatory monocytosis in CVD and stroke

Inflammatory monocytes are the major cellular component in atherosclerotic plaque [47]. Accumulation of activated immune cells, including inflammatory monocytes and macrophages, and T lymphocytes in the vessel wall produce inflammatory cytokines and facilitate vascular inflammation.

Inflammatory monocytes may contribute to vascular inflammation not only by producing inflammatory cytokines, but also via CD40-mediated T cell activation. It was reported that CD40-CD40 ligand (CD40L) signaling, a T cell co-stimulatory receptor-ligand pair, plays a crucial role in atherosclerosis [48]. The action of T cells in atherosclerosis is similar to a CD4^+^ T helper cell 1 (Th1)-mediated hypersensitivity reaction, which might use ox-LDL as a possible auto-antigenic stimulus [49]. In human atherosclerotic lesions, CD40–CD40L are co-localized with epitopes of ox-LDL, scavenger receptor A (a mediator of foam cell formation), and CD16 [50]. CD40 is a TNF receptor superfamily 5 member and is expressed in monocytes, macrophages, dendritic cells. CD40 ligand is found on CD4^+^ T cells and platelets in both secreted and membrane bound forms. CD40-CD40L expression on platelets enhances platelet activation and thrombosis [51]. CD40 and CD40L are both expressed on endothelial cells and vascular smooth muscle cells. Either CD40 or CD40L deficiency in *ApoE*^-/-^ mice abrogated atherosclerosis by increasing the extracellular matrix and promoting M2 macrophage polarization [52].

Classical CD14^+^ monocytes are critical for clearance of LDL, whereas CD16^+^ monocytes including intermediate and nonclassical monocytes have higher expression levels of major histocompatibility complex class II (MHC-II) and higher capacity to uptake ox-LDL [53]. CD40 signaling induced the expression of adhesion molecules, matrix metalloproteinases and proinflammatory cytokines in macrophages and foam cell formation [54]. It was reported that monoclonal antibodies against CD40L reduced atherosclerosis rendered thromboembolic complications [55]. Thus, antagonizing CD40 signaling or suppressing CD40 expression might be future therapeutic alternatives for human CVD.

Similarly, monocytes are the major infiltrating immune cells in the ischemic brain in stroke. Monocyte infiltration is one of the earliest cellular response in stroke. It occurs 4 hours after stroke and reaches maximum infiltration in 7 days [56]. Inflammation accompanying stroke plays an important role in secondary ischemic injury [57]. Infiltrated inflammatory cells can produce ROS, inflammatory cytokines and matrix metalloproteinase, inducing neuron injury directly or indirectly by inducing blood brain barrier (BBB) disruption, which can lead to edema, cerebral hemorrhage and a vicious circle of continuous influx of myeloid cells. However, the inflammatory effects on the stroke process can be detrimental or protective, depending on the immune cell types, numbers and duration. A recently published paper indirectly supported the detrimental role of monocytes in stroke [58]. Bone marrow transplantation from *ApoE*^-/-^*CD36*^-/-^ (mostly expressed in monocytes) donor mice to *ApoE*^-/-^ recipient mice decreased infarction volume and neurological deficits after stroke. But the roles of different monocyte subsets in the pathogenesis of stroke remain unclear. Ly6C^+^ monocytes have been proven to be responsible for many central nervous system diseases like autoimmune multiple sclerosis [59] and infectious encephalitis caused by West Nile virus [60]. The chemokine receptor CCR2 deficiency, which is the main chemokine receptor for recruiting Ly6C^+^ monocytes, attenuates infarction size and neurological deficit after stroke in the transient middle cerebral artery occlusion (tMCAO) stroke mouse model, accompanying significantly reduced monocyte and neutrophil infiltration [61]. Also, there is a report pointing out that the Ly6C^-^ macrophages differentiated from infiltrating Ly6C^+^ monocytes are critical for preventing hemorrhagic infarct transformation in both the tMCAO and the photo thrombosis induced permanent stroke models [62]. However, Ly6C^+^ monocyte depletion by clondronate liposome or by bone marrow transplantation from *CCR2*^-/-^ donor mice to wild type recipient mice showed dramatically increased hemorrhage occurrence rates without changing infarction volume and neurological function. The reason why the same CCR2 deficiency mice display different results is unknown, it may be due to different mouse breeding methods since pure knockout mouse cross-breeding for several generations may lead to gene changes, which may compensate for the designated gene defect. To determine the roles of different monocyte subsets in stroke pathogenesis, more experiments should be conducted in the context of normal or combined disease settings like hyperlipidemia and hyperhomocysteinemia.

## Conclusion

To date, many studies have demonstrated the key roles of inflammatory and anti-inflammatory monocytes in response to inflammation or steady state in mouse models. Inflammatory monocyte subset is a valuable biomarker for human inflammatory diseases, including cardiovascular diseases. Understanding the mechanism of monocyte differentiation will likely provide a potential therapeutic target for inflammatory monocytosis.

## Competing interests

The authors declare that they have no competing interests.

## Authors’ contributions

YJ generated figures and tables and drafted manuscript. ZL contributed on text for inflammatory monocytosis in stroke and partially monocyte differentiation. YC contributed on scientific discussion and editing scientific writing and English. X-FY contributed to manuscript design and final review. WH is responsible for the manuscript design and final writing. All authors read and approved the final manuscript.
